# Preserved cardiac function by vinculin enhances glucose oxidation and extends health- and life-span

**DOI:** 10.1063/1.5019592

**Published:** 2018-07-17

**Authors:** Ayla O. Sessions, Peter Min, Thekla Cordes, Barry J. Weickert, Ajit S. Divakaruni, Anne N. Murphy, Christian M. Metallo, Adam J. Engler

**Affiliations:** 1 Biomedical Sciences Program, UC San Diego, La Jolla, California 92093, USA; 2 Department of Bioengineering, UC San Diego, La Jolla, California 92093, USA; 3 Department of Pharmacology, UC San Diego, La Jolla, California 92093, USA; 4 Sanford Consortium for Regenerative Medicine, La Jolla, California 92037, USA

## Abstract

Despite limited regenerative capacity as we age, cardiomyocytes maintain their function in part through compensatory mechanisms, e.g., Vinculin reinforcement of intercalated discs in aged organisms. This mechanism, which is conserved from flies to non-human primates, creates a more crystalline sarcomere lattice that extends lifespan, but systemic connections between the cardiac sarcomere structure and lifespan extension are not apparent. Using the rapidly aging fly system, we found that cardiac-specific Vinculin-overexpression [Vinculin heart-enhanced (*VincHE*)] increases heart contractility, maximal cardiac mitochondrial respiration, and organismal fitness with age. Systemic metabolism also dramatically changed with age and *VincHE*; steady state sugar concentrations, as well as aerobic glucose metabolism, increase in *VincHE* and suggest enhanced energy substrate utilization with increased cardiac performance. When cardiac stress was induced with the complex I inhibitor rotenone, *VincHE* hearts sustain contractions unlike controls. This work establishes a new link between the cardiac cytoskeleton and systemic glucose utilization and protects mitochondrial function from external stress.

NOMENCLATURE

ANOVA: Analysis of variance
CVD: Cardiovascular disease
GC-MS: Gas chromatography-mass spectrometry
ID: Intercalated disc
MSTFA: 2,2,2-trifluoro-*N*-methyl-*N*-trimethylsilyl-acetamide
MTBSTFA: *N*-tertbutyldimethylsilyl-*N*-methyltrifluoroacetamide
OCR: Oxygen consumption rate
PCA: Principal component analysis
UAS: Upstream activation sequence
*VincHE*: Vinculin heart-enhanced flies

## INTRODUCTION

Aging is a complex process associated with the progressive decline of physiological function and is a significant risk factor for cardiovascular disease (CVD); as human lifespan increases, CVD risk also increases.^1^ With age, the heart remodels extensively, altering extracellular matrix and intracellular structures that decrease elasticity and contractile compliance.^2–4^ Remodeling creates a feedback loop that further increases systolic pressure and afterload.^5,6^ Remodeling also results in left ventricular wall thickening and impaired myocardial performance.^7,8^ Counterbalancing these negative processes are compensatory mechanisms that reinforce sarcomeres and the sarcolemma to help manage age-associated increases in heart wall strain.

We have identified one such compensatory mechanism where Vinculin-mediated cytoskeletal reinforcement at the intercalated disc (ID) and costamere improves force production and myofibril geometry.^9^ Vinculin is a critical ID linker protein involved in both sensing and translating sarcomeric contraction into cell shortening. In aged patients with heart failure, Vinculin undergoes extensive remodeling^10,11^ that is reversed when the heart is unloaded.^12,13^ Using a multi-model approach, we identified that cardiac Vinculin upregulation with age is a conserved mechanism across non-human primate, rat, and fly models independent of cardiovascular disease. Further interrogation in *Drosophila melanogaster—*the fruit fly*—*using cardiac specific overexpression of Vinculin [Vinculin heart-enhanced (*VincHE*)] revealed increased Vinculin recruitment to both the ID and costamere, which improved cardiac contractility via longitudinal increases in force production, lateral increases in myofibrillar anchoring, and improved sarcomere lattice geometry. Under significant load, Vinculin overexpression preserved contractility,^9^ suggesting that Vinculin upregulation is a conserved mechanism to counterbalance age-associated stress and extend organismal lifespan. Despite these observations, mechanisms connecting improved sarcomere crystallinity and longevity are unclear. Both *Drosophila* and *Caenorhabditis elegans* models have been used in recent years to screen for lifespan-altering genes.^14,15^ However, these early screens focus on organismal differences rather than tissue specific changes that could regulate systemic effects. Changes to integral organs such as the heart, implicated in the delivery of exogenous factors, nutrients, and O_2_, have yet to clearly describe the mechanisms that support such correlations.

Of the systemic changes that could be influenced by cardiac output, metabolism is one function that directly impacts lifespan. Early work in *Drosophila* noted a link between a temperature-induced decrease in metabolism and a significant increase in lifespan.^16^ In addition, metabolic profiling of longevity-selected *Drosophila* established clear differences in steady-state metabolite composition at all ages compared to age-matched controls.^17^ This mirrors similar data in *C. elegans*^18^ and mice.^19^ Caloric restriction studies in *Drosophila*^20^ and mice^19^ provide another link between organismal metabolism and longevity, suggesting not only that caloric restriction causes increased lifespan, but that slowing the changes in the metabolome of flies with age conferred these systemic benefits. These findings largely focus on identifying metabolic biomarkers of aging and longevity as opposed to how local organ changes could ultimately skew organismal metabolism, e.g., improved cardiac function from a more robust sarcomere. Since the heart requires a continuous supply of energy to function yet stores a minimal amount locally, any changes in contractility and energy demand, either local or systemic, will cause significant changes in energy homeostasis and metabolism. Improving heart physiology could alter systemic nutrient delivery, thus having a significant impact on healthspan and activity levels in advanced age.

These data from longevity selection and cardiac-restricted Vinculin-overexpression together suggest a unique link: we hypothesize that the increased contractile capacity and sustained function of Vinculin overexpressing hearts (*VincHE*) with age increase organismal fitness through improvements to systemic energy metabolism, thereby increasing longevity. We found that a cardiac-specific increase in Vinculin expression increases basal contractility, which helps sustain cardiac respiration with age and alters sugar utilization and glucose oxidation through the tricarboxylic acid (TCA) cycle. These modifications allow *VincHE* hearts to perform better under mitochondrial stress. Thus, these data for the first time establish a link between cardiomyocyte cytoskeletal remodeling and systemic metabolism and function.

## RESULTS

### Improved contractility from cardiac-specific Vinculin over-expression improves fitness, extends lifespan, and sustains cardiac respiration with age

**FIG. 1.**
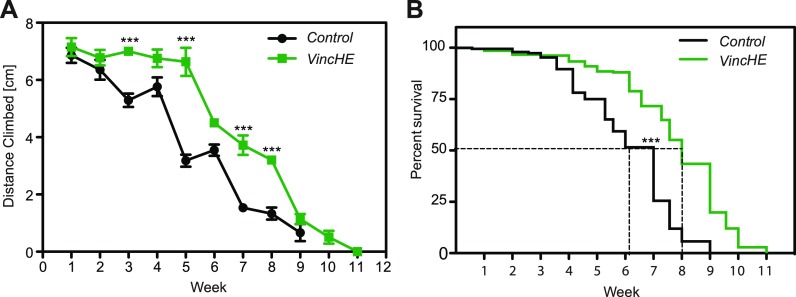
Increased climbing ability and significant increases in lifespan are observed in *VincHE* flies. (a) Rapid Iterative Negative Geotaxis (RING) assay described in detail in the methods was performed weekly over the entire lifespan of each genotype, and the climbing distance over an interval of 5 s was recorded for each fly. n = 250 at the start of the experiment. Data represent mean ± SEM. All negative geotaxis data were analyzed with 2-way-ANOVA with post hoc Bonferroni test. *** p < 0.001. (b) Survival curves for *VincHE* and controls in the RING assay show median survival indicated by the dashed black line for each genotype. ***p < 0.0001.

The *D. melanogaster* strain *w^1118^* was genetically modified to overexpress Vinculin in their hearts via cardiac-specific tinHE-Gal4 driver (tinman heart enhancer) in conjunction with upstream activation sequence (UAS)-inducible *Vinculin* [Vinculin heart-enhanced (*VincHE*)]. The resulting cardiac-specific over-expression of *VincHE* hearts resulted in higher basal wall shortening velocities, increased velocity under viscous load, and a less severe load dependent decline in fractional shortening, i.e., the ratio of tube diameter change to its diastolic diameter.^9^ While evidence supports a link between cardiovascular function and aerobic exercise capacity^21^ as well as its age-associated decline,^22–24^ it is not clear to what extent and how systemic changes result from cardiac-specific sarcomere changes. Thus, we assessed the fitness of *VincHE* flies as a function of age using the rapid iterative negative geotaxis (RING) assay, which assesses the natural climbing response in flies.^25,26^ When tracked over their lifespan and as a function of genotype, *VincHE* flies exhibited increased climbing ability between the ages of 3 and 8 weeks compared to controls [Fig. 1(a)] until the median lifespan was surpassed [Fig. 1(b)]. Forced climbing significantly increases energy demand, so these data from flies with cardiac overexpression of Vinculin suggest that benefits are not only local to the heart and that it significantly increases the activity in aging organisms; it should be noted that activity progressively declines independent of Vinculin expression but that there is prolonged activity for *VincHE* flies consistent with prolonged lifespan.

**FIG. 2.**
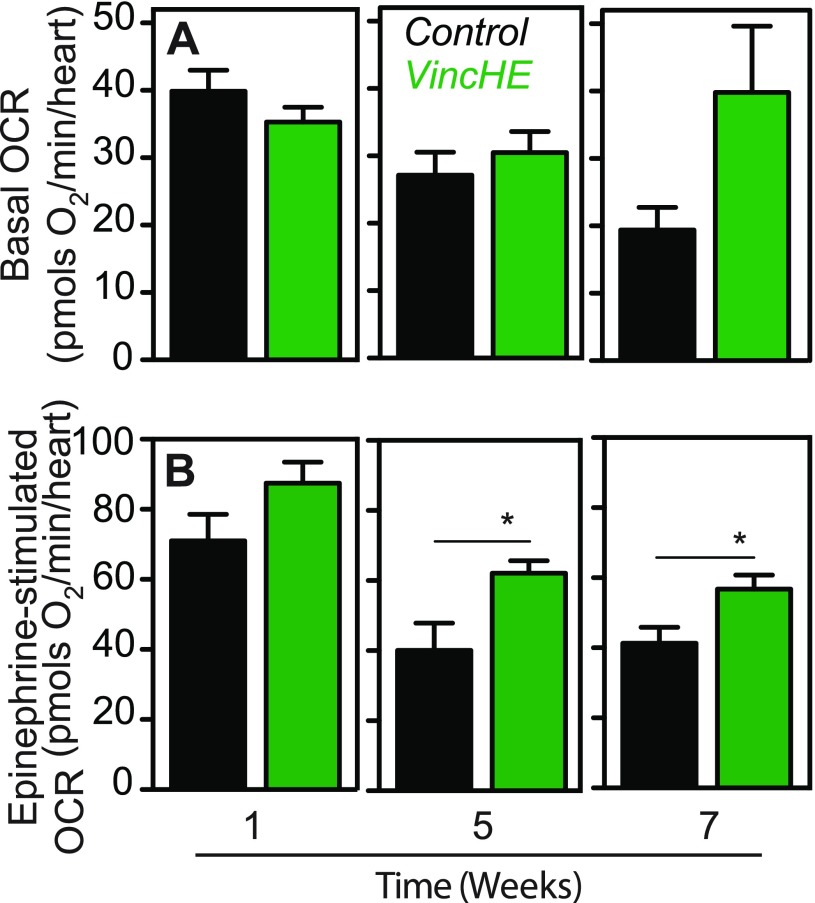
*VincHE* hearts exhibit sustained oxygen consumption due to contraction and increased maximal respiration under cardiovascular stress with age. (a) Oxygen consumption rates (OCR) were calculated as the difference between the endogenous (basal) and non-mitochondrial respiration (after addition of rotenone and antimycin A) at the given ages. 12 < n < 20 for all samples in biological triplicates. (b) Stimulated OCR of *VincHE* and control hearts were measured after the addition of 1 mM epinephrine and corrected for background rates determined with the addition of rotenone and antimycin A. Data represented as mean ± SEM. 12 < n < 20 for all samples in biological triplicates. All respiration data were analyzed with a two-tailed Student's t-test and Mann-Whitney post-test where *p < 0.05. Fly ages are indicated on the x-axis.

Since alterations to the cardiac sarcomere lattice structure have been postulated to change the contractile efficiency,^27^ we next determined whether *VincHE* flies had altered cardiomyocyte bioenergetics by adapting the Seahorse XF24 extracellular flux analyzer to measure respiration of individual, surgically exposed and contracting hearts. Oxygen consumption rate (OCR) was proportional to the number of hearts assayed, though we obtained sufficient signal with a single heart, allowing us to measure fly-to-fly variation [Fig. S1(a)]. Regardless, basal OCR of *VincHE* flies sustained respiration with age, whereas control flies lacking this over-expression experienced a decline in OCR with age [Fig. 2(a)]. Next, we utilized epinephrine, a beta-adrenergic receptor agonist, as a stress to increase contraction and stimulate respiration [Fig. S1(b), right]. After epinephrine exposure, *VincHE* hearts exhibit a profound increase in respiration, and this increase was sustained with age, whereas OCRs in control hearts significantly decreased [Fig. 2(b)]. These data suggest that the preserved myofibril lattice order in *VincHE* flies^9^ is associated with an increase in the mitochondrial respiratory capacity and the ability to handle contractile stress with age. While a causal relationship between Vinculin and OCR has not been previously established to our knowledge, these data are consistent with improved cardiac function resulting from pharmacologically mediated improvements in mitochondrial respiration.^28^

### Oxidative glucose metabolism is increased in *VincHE* flies

**FIG. 3.**
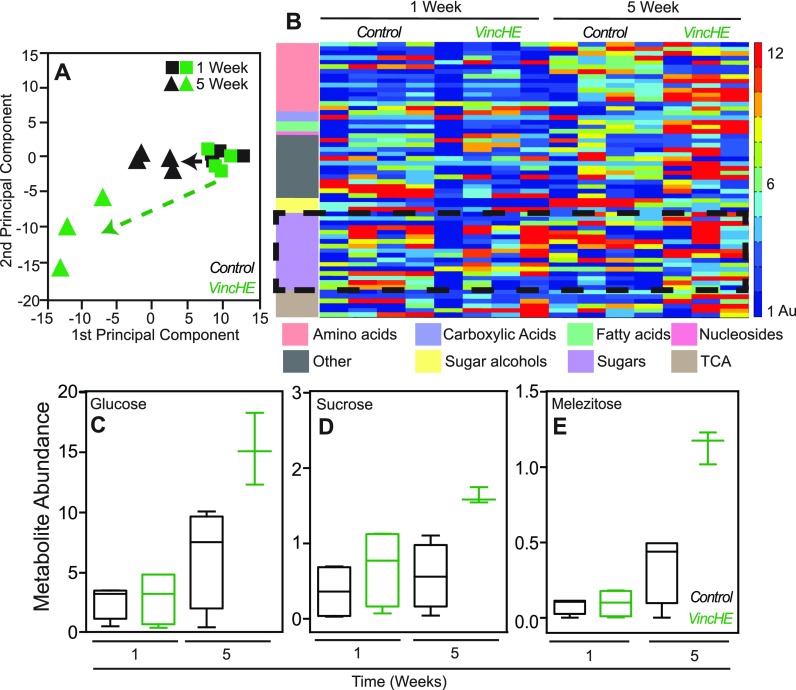
Whole fly GC-MS metabolic profiling reveals metabolic changes in energy metabolism *in VincHE* with age. (a) Principal component analysis of biological replicates scaling expression values is plotted for given genotypes (green for *VincHE* and black for *control*) and ages (squares for 1 week and triangles for 5 weeks). (b) Heat-map visualization of changes in metabolites identified in whole fly GC-MS analysis normalized to maximal intensity detected across samples and scaled on an expression scale of 1–12 for each genotype and age specified. Metabolites were sorted by ontology and color coded to the left with colors and associated ontology specified at the bottom. Dashed black box highlights sugar ontology. (c) Sucrose, (d) glucose, and (e) melezitose abundance plotted for each genotype at 1 or 5 weeks of adulthood. n = 4 with each five flies per condition. Data were analyzed, and metabolites identified using MetaboliteDetectore software. Statistical significance calculated by 2-way ANOVA with post-hoc Dunn's test. * p < 0.05.

To identify specific metabolites that could contribute to improved stress tolerance and fitness with age in whole *VincHE* flies, we profiled metabolism in a non-targeted manner using gas chromatography–mass spectrometry (GC-MS). Of the 280 detected features, 61 had identifiable metabolic signatures. Principal component analysis (PCA) revealed differential metabolite abundances clustering as a function of both genotype and age [Fig. 3(a)], indicating that significant systemic metabolic changes occur as a result of Vinculin-overexpression in the heart. Ontological clustering showed significant accumulation for most of the identified metabolites in aged *VincHE* flies compared to controls and specific clustering for sugars and amino acids [Fig. 3(b)]. First, sugars such as glucose and sucrose had preferential accumulation in moderately aged *VincHE* flies [Figs. 3(c) and 3(d)], consistent with data suggesting glucose accumulation in longevity selected flies.^17^ In addition, we detected significantly increased levels of melezitose with age in *VincHE* flies [Fig. 3(e)]. This trisaccharide sugar is known to reduce osmotic stress in insects and prolong lifespan in *Drosophila.*^29^ Second, amino acid balance is thought to play an important role in diet restriction-mediated lifespan extension^30^ and in Target of Rapamycin (TOR) pathway activation;^31^ we found that the amino acid metabolite cluster was significantly increased with age in *VincHE* flies [Fig. 3(b)]. Homoserine, a metabolite associated with methionine, threonine, and isoleucine metabolism, exhibited significant accumulation in *VincHE* at 5 weeks [Fig. S2(a)], which could influence methionine availability; methionine restriction extends lifespan in mouse, rat, and fly.^32–34^ Conversely, additional lysine increases *Drosophila* longevity,^17^ and as longevity selected flies, *VincHE* flies exhibit increased lysine levels with age [Fig. S2(b)]. These data strongly indicate that alteration of metabolite levels or efficiency of sugar utilization may correlate with increased lifespan and healthspan.

Steady-state metabolomic profiling implicated accumulation of sugar metabolites, e.g., glucose, with *VincHE* flies, so we next analyzed incorporation of glucose into downstream metabolic pathways in a more targeted manner using uniformly ^13^C labeled glucose ([U-^13^C_6_]glucose). Flies were initially fasted for 12 h and then allowed to ingest labeled glucose; fasting ensured uniform uptake while not affecting fly mortality [Fig. S3(a)]. To demonstrate ^13^C incorporation from glucose into downstream metabolites, we performed a time-course and analyzed metabolism of control flies offered labeled glucose. ^13^C incorporation, calculated as mole percent enrichment (MPE), into pyruvate and TCA cycle intermediates increased linearly over time with robust labeling after 1 h post feeding [Fig. S3(b)].

**FIG. 4.**
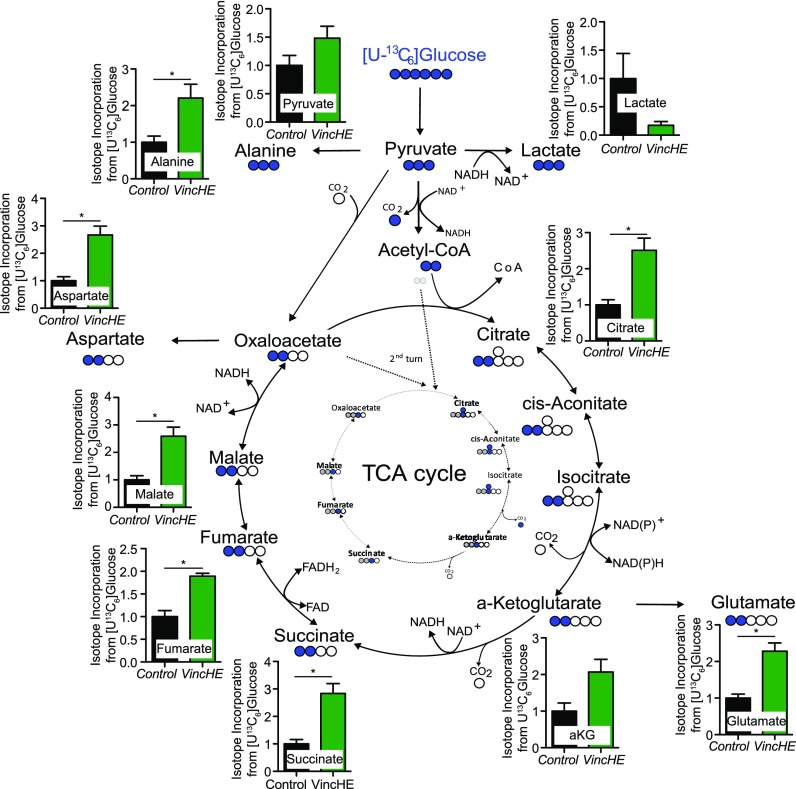
*VincHE* flies exhibit increased ability to metabolize glucose via incorporation into the TCA cycle. Mole percent enrichment (MPE) of [U-^13^C_6_]glucose shown for pyruvate, lactate, TCA cycle intermediates, and related amino acids relative to control conditions at 1 week of adulthood. Flies were fasted for 12 h before offering labeled glucose. The metabolic map depicts incorporation of [^13^C]glucose-derived carbons into downstream metabolites. Open circles depict ^12^C, closed circles ^13^C atoms (blue first turn, grey second turn of the TCA cycle). Data represented as mean ± SEM. n = 4 with five flies per condition. Statistical significance calculated by non-parametric Students t-test with Mann Whitney post-test. * p < 0.05.

^13^C enrichment in metabolites was quantified after adult 1-week *VincHE* and control flies consumed labeled glucose for 1 h after fasting; 1-week flies were chosen to focus on flux changes specifically associated with the genotype as opposed to phenotypic changes caused by differential aging (i.e., survival and activity were significantly different at 5-weeks). Relative MPE values show that *VincHE* flies exhibit significantly increased glucose incorporation into pyruvate, TCA cycle intermediates (citrate, a-ketoglutarate, succinate, fumarate, and malate), and related amino acids (alanine, glutamate, and aspartate) compared to control conditions (Fig. 4). On the other hand, MPE of lactate was decreased in *VincHE* flies, indicating that vinculin overexpression redirects glucose-derived carbons away from lactate and into the TCA cycle. Metabolite abundance (instead of flux) also mirrors MPE after the overnight fasting [Fig. S4(a)] and further supports the conclusion that mitochondrial glucose oxidation is increased in *VincHE*. Conversely, abundance decreased for urea [Fig. S4(b)], a known marker of aging which can cause cardiovascular disease,^35,36^ and for branched-chain (valine, leucine, and isoleucine) and other amino acids (threonine, phenylalanine, tyrosine, and tryptophan) in *VincHE* flies [Fig. S4(c)]. These data are consistent with observations where high urea and amino acid concentrations have been linked to diabetes, insulin resistance, and cardiovascular disease.^37–39^ Taken together, these data strongly suggest that altered systemic glucose metabolism is associated with maintenance of the sarcomere structure in the heart.

### *VincHE* flies are resistant to mitochondrial stress and protected from adverse cytoskeletal remodeling

**FIG. 5.**
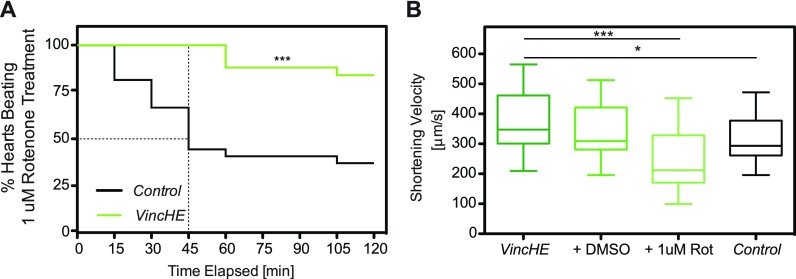
*VincHE* hearts exhibit increased resistance to mitochondrial stress when subjected to rotenone treatment compared to controls and maintain physiological heart wall velocities. (a) Percent of hearts contracting with fractional shortening >5% were plotted vs time for *VincHE* and control hearts exposed to mitochondrial stressor 1 *μ*M rotenone. n = 25 for 1 week old adult hearts. Data were analyzed by Log-rank Mantel Cox test. *** p < 0.0001. (b) Heart wall shortening (contraction) velocities were assessed for *VincHE* hearts in hemolymph or treated with either 0.05% DMSO or 1 *μ*M rotenone in 0.05% DMSO for 30 min (green) and untreated controls (black). All data were analyzed with a one-way ANOVA and Tukey post-test. n > 15. * p < 0.05, *** p < 0.001.

To stress bioenergetics in *VincHE* flies, rotenone, a mitochondrial complex I inhibitor, was added to hemolymph and contraction monitored. Adult *VincHE* hearts maintained rhythmic contraction despite mitochondrial stress, whereas contraction arrested in >50% of controls by 45 min [Fig. 5(a)]; dimethyl sulfoxide (DMSO) alone exhibited no significant effects [Fig. S5(a)]. Since *VincHE* hearts could withstand prolonged stress, heart wall velocity was assessed by m-mode imaging before and after 1 *μ*M rotenone treatment. Untreated *VincHE* hearts exhibited higher basal shortening velocity over controls as reported,^9^ but during stress, *VincHE* hearts exhibited shortening velocities within the physiological range compared to untreated controls [Fig. 5(b)]. These findings indicate the protective effect of Vinculin, so we next sought to understand how mitochondrial inhibition affects the cell cytoskeleton.

Given difficulties to assess acute remodeling *in situ*, we examined remodeling in the immortalized *Drosophila* cell line S2R+, which naturally overexpresses Vinculin versus other *Drosophila* cell lines.^40^ S2R+ cells were treated with 2 *μ*M rotenone and after 15 min, observable morphological changes occurred. S2R+ cells normally adhere and spread out onto the tissue culture plate forming lamellipodia [Fig. S5(b)]; however, blocking mitochondrial respiration with rotenone caused cells to shrink and form filopodia-like protrusions [Fig. S5(c), arrows]. The treatments not only led to a significant decrease in cell size [Fig. S5(d)] but also a decrease in βPS integrin intensity per cell [Fig. S5(e)], which may indicate focal adhesion disassembly or, at the very least, indicate cytoskeletal reorganization and decreased adhesive ability. Together, these results provide further evidence that adult *VincHE* flies exhibit enhanced mitochondrial glucose metabolism as well as greater resistance to mitochondrial stress through a more organized cytoskeleton.

## DISCUSSION

These data demonstrate that fly heart-specific Vinculin over-expression increases contractility, sustaining cardiac respiration with age or under conditions of mechanical stress. The resulting cardiac benefits, even in aged *VincHE* cohorts, increase organismal fitness with age. This may occur in part due to more efficient use of nutrients in *VincHE* flies, as aerobic oxidation of glucose in mitochondria yields significantly more adenosine triphosphate (ATP) compared to glycolysis. To this end, isotope tracing demonstrated increased glucose oxidation by mitochondria in *VincHE* flies as shown by increased ^13^C incorporation from labeled glucose in TCA cycle intermediates after feeding. Overall, cytoskeletal reinforcement in *VincHE* hearts may serve as the underlying mechanism that allows them to resist mitochondrial stress and maintain rhythmic contraction. These findings are the first to document that cardiac-restricted cytoskeletal remodeling results in a systemic metabolic response. Despite this specificity, our perturbation led to organismal metabolite accumulation similar to longevity selected organisms and to the identification of a systemic mechanism, i.e., enhanced aerobic glucose metabolism in adult flies, which could explain significant increases in lifespan and healthspan.

### Cytoskeletal remodeling maintains fitness and respiration in aged organisms

Heart aging has been classically associated with remodeling where more extracellular matrix is expressed, elasticity is decreased, and compliance is limited;^2–4^ a negative feedback loop further remodels the heart as afterload; and systolic pressure^5,6^ causes wall thickening and impairs myocardial contraction.^7,8^ Although typical age-associated fibrosis is limited in *Drosophila*^41^ making compensatory mechanisms easier to study,^42,43^ these mechanisms can still be observed in the hearts of higher organisms.^44,45^ Age-associated compensatory mechanisms are not limited to the heart as other tissues, including skeletal muscle, undergo significant remodeling with age, e.g., sarcopenia.^46,47^ Beyond these specific examples, additional recent evidence suggests that many counterbalancing and underappreciated mechanisms may attempt to maintain organ function^44^ and that mechanisms can be organ- and protein-specific such as Vinculin.^9^

To the best of our knowledge, our data are the first to show that cardiac-specific overexpression of a cytoskeletal protein, which is naturally upregulated in many species and strains with age and results in local improvements in sarcomere organization,^9^ not only extends lifespan but also improves healthspan, e.g., measures of fitness with age. While such assays measure a multitude of behaviors, including locomotor function, climbing ability, and escape reflex, they also offer significant information on age-dependent decline in skeletal muscle function.^25,48^ Other studies that restrict diet^49^ or provide an exercise regimen^22^ observe temporary changes in function or locomotion but not both, which suggests that transgenic overexpression or higher basal expression of a heart cytoskeletal protein with age could improve organismal fitness by maintaining sufficient respiratory capacity for organs with high metabolic demand, e.g., flight muscles. Improved locomotion may also be explained by our previous observation that more Vinculin in the intercalated discs causes myofibrils that terminate at its junction to be more crystalline. A more regular sarcomere lattice has higher power production from faster actin-myosin cross-bridge cycling,^9,50^ which could result in higher cardiac respiration. While basal oxygen flux was not significantly different *for* VincHE flies (data not shown), *VincHE* hearts stressed by the adrenergic agonist epinephrine increase the maximal OCR and sustain it with age. Moreover, when hearts are stressed by pumping a more viscous fluid, we found that they exhibit increased contractile velocity with elevated afterload. These data for the first time suggest a link between improved contractions from cytoskeletal remodeling and respiration, which may result in better-perfused organs with age as suggested by OCR measurements.

### Robust cardiac sarcomeres alter energy requirements and protect cells from stress

When thin and thick filaments are in better registry with one another, i.e., through cardiac-restricted Vinculin overexpression at the intercalated discs,^9^ they cycle between bound and unbound states faster and have higher binding probabilities.^27^ Better cycling alters energy requirements in the heart, but it was uncertain if similar changes could be observed systemically. Untargeted whole fly GC-MS revealed significant energy metabolite accumulation for *VincHE* flies in all ontological clusters with age. These increased sugar concentrations in *VincHE* flies mirror metabolic profiling of longevity selected flies^17,51^ and caloric and amino acid restriction, which also affects lifespan.^30,49,52–54^ Interestingly, sugar accumulation resulting from more efficient metabolism via cytoskeletal remodeling in the heart appears to be beneficial, but its utilization in a high sugar diet is not. A high sugar diet decreases heart function, induces pericardin accumulation—a fibrosis-like collagen, and shortens lifespan.^41^ *VincHE* flies also exhibited significant amino acid accumulation, specifically homoserine and lysine. Homoserine is a precursor for methionine, and it is well documented that methionine restriction extends lifespan in several model organisms.^32–34^ Methionine metabolism affects downstream polyamines, cysteine, and glutathione synthesis and DNA methylation via a transsulfation pathway, so its accumulation in *VincHE* flies could have significant impact on any of these downstream pathways.^55^ Thus, our whole fly metabolic profiling suggest that *both* age, consistent with other aging profiles for *Drosophila,*^56,57^ *and* cardiac-specific genetic perturbation affect systemic metabolism consistent with the effects of at least one other cardiac perturbation on organismal metabolism.^58,59^ It is not known why some of these other metabolic changes have such a significant impact on organismal lifespan, but here, we show a strong correlation between vinculin-mediated improvements in systemic metabolic efficiency and organismal fitness, which implies a new paradigm for cytoskeletal-mediated changes in metabolism.

Glucose tracing further demonstrated that *VincHE* flies have increased glucose-derived carbon flux into the TCA cycle and decreased lactate levels. The heart predominantly uses fatty acids for ATP production under normal circumstances but will switch to fast glucose metabolism for ATP production during stress.^60^ We observed that whole *VincHE* flies could redirect carbons into the TCA cycle for ATP production compared to controls, which suggests Vinculin expression in the heart could better equip the fly to handle external stressors associated with aging. This unique interaction between cardiac-restricted Vinculin overexpression and systemic mitochondrial ATP production was further validated by stressing cells with the mitochondrial complex I inhibitor rotenone.^61^ We found that improved cardiac sarcomere lattice organization in the *VincHE* flies prevented rotenone-mediated reduction in the fraction of contractile hearts and also maintained shortening velocities within a physiological range. Conversely, rotenone treatment of *Drosophila* S2R+ cells, despite higher basal Vinculin expression than other *Drosophila* cell lines and tissues,^40^ exhibited significant cytoskeletal depolymerization. S2R+ cells contain focal adhesions, not sarcomeres, and so, these data are consistent with similar observations of disassembly^62^ but with Vinculin overexpression to compensate for rotenone treatment.^63^ Thus, in myocytes, it may be plausible that protection may be due to improved cross-bridge cycling,^27^ i.e., lower ATP demands, and a more stabilized cytoskeleton.

In addition to preferential glucose oxidation in *VincHE* flies, metabolic studies also revealed decreased abundance of metabolites associated with aging, insulin sensitivity, and cardiovascular disease.^37–39^ Amino acids such as branched-chain amino acids (BCAAs) as well as tyrosine and phenylalanine are associated with diabetes and aging, and these were all significantly decreased in *VincHE* flies compared to controls at 1 week. While integral for proper function, elevated circulating BCAAs are found in obese individuals and indicate worse metabolic health and future insulin resistance.^39,64–66^ BCAAs have also been implicated in cardiovascular disease and heart failure^67–69^ indicating that cardiac improvements in *VincHE* flies may exhibit a reversal of metabolic profiles associated with adverse function. These findings implicate not only increased glucose metabolism but also decreased metabolite abundances typically associated with insulin resistance and diabetes which is consistent with earlier work in *Drosophila* that suggested insulin-IGF (Insulin Growth Factor) signaling influences age-dependent cardiac physiology and lifespan.^70^ These data together identify how altered glucose metabolism, increased mitochondrial function under stress, and insulin signaling could collectively work to increase energy stores under stress, sustain cardiovascular function with age, and improve longevity. We believe that our data is the first to link a heart-specific cytoskeletal change to a systemic increase in glucose flux and decrease essential amino acids, which better buffers against age-related remodeling of these targets associated with impaired cardiac function.

These data overall suggest for the first time that Vinculin-mediated lifespan extension could be attributed not only to heart-specific improvements in performance but also to systemic metabolic benefits resulting from maintaining cardiac output with age. These observations further suggest specific targets, whether in the cytoskeleton or their downstream metabolic mediators, that could potentially improve fitness in higher organisms.

## METHODS

### *Drosophila* lines, husbandry, and culture conditions

Fly line UAS-Vinculin (#21870) was obtained from the Bloomington *Drosophila* Stock Center at Indiana University (NIH P40OD018537). Cardiac-specific gene regulation was achieved with the Gal4-UAS system as described previously.^71^ UAS-transgenic males were crossed with virgin female *tinHE-Gal4* flies using the GAL4-UAS system as previously described.^72,73^ Female progeny of tinHE-Gal4 and *w^1118^* served as control. Proper insertion of both constructs was validated using heart-specific gene expression analysis.^9^ Flies were raised on standard agar-containing food at 25 °C. Food was changed every third day. Ethics approval is not required for the work with invertebrates.

### *Drosophila* microsurgeries

Preparation of the *Drosophila* hearts high speed imaging and respiration measurements were performed as previously described,^74^ and did not affect heart function.^9^ Adult female flies were briefly exposed to 5 psi CO_2_ (<1 min), then anesthetized with Fly Nap (Carolina Biological) for 5 min, and their dorsal side secured to a 35 mm petri dish. The beating heart was exposed by incisions to remove head, thorax, and the ventral abdominal cuticle. Flies were submerged in an artificial oxygenated hemolymph at 25 °C.^75^ Internal organs and debris above the beating heart were all carefully aspirated with a micropipette, making sure to not perturb the heart as that could result in a hypercontractile state. Rhythmic contraction can be sustained for hours with regular oxygenation and hemolymph changes, but all experiments were performed within 1 h of microsurgery.

### Activity and lifespan assessments

Climbing ability was assessed as previously described with the Rapid Iterative Negative Geotaxis (RING) assay.^25,26^ 200 flies per genotype were placed into vials of 25 flies each on standard agar containing food. Each week, on the day prior to measurement, flies were briefly anesthetized with CO_2_, placed into a fresh vial, and allowed to recover overnight. During the experiment, flies were transferred into new 10 cm flat bottom polypropylene digestion tubes (Environmental Express) without CO_2_ to prevent any anesthesia agent effects on performance. Four tubes at a time were placed side by side into the RING apparatus.^26^ After acclimating for 20 min, 5 sets of negative geotaxis at 1-min intervals were recorded where flies were forced to the bottom of their tubes and then allowed to climb. Still images 5 s after each tap were acquired, and distance traveled was measured. Mean height climbed between 0 and 9 cm was calculated for each genotype, and two-way analysis of variance (ANOVA) with a Bonferroni post-test was performed to assess significance at each time-point over their entire lifespan. Flies were transferred into new food vials for continued testing throughout their lifespan. Lifespan and deaths were also recorded every 2 days when food was changed to construct a Kaplan-Meier plot. Females were also separately collected in groups of 25 flies in each vial, kept at 25 °C, and dead flies counted every 2 days after transfer onto new standard agar-containing food. Each experiment consisted of 200 flies.

### Seahorse oxygen consumption assay

A method was optimized to use the Seahorse XF24 extracellular flux analyzer and associated consumables (Agilent) to correlate the O_2_ consumption rate (OCR) with cardiac function by measuring rates of oxygen consumption of intact hearts. Rigorously cleaned *Drosophila* hearts attached to a trimmed cuticle were placed at 1 heart/well into a 24-well islet capture plate using tweezers. Tissue was secured to the center of the well using a small amount of vacuum grease. 400 *μ*l of hemolymph described above with 5 mM phenol red was added to each well. A capture screen was placed over each recessed well to keep the tissue in place. All hearts were checked for contractility before the start of the assay. OCR was assayed through the injection of a series of compounds; basal respiration was assessed prior to the injection of any pharmacological compounds. Although exhaustive attempts to measure state 4 (with oligomycin) and maximal carbonyl cyanide-4-(trifluoromethoxy)phenylhydrazone (FCCP)-induced uncoupled respiration were made by testing differing concentrations and incubation times, the lack of appropriate changes in OCR suggested that oligomycin and FCCP did not permeate the heart tissue. Therefore, these data are not shown. However, non-mitochondrial OCR/instrument background was able to be measured after addition of 1 *μ*M rotenone and 4 *μ*M antimycin A. Sixteen loops of mixing and 2 min measurements were required after the addition of rotenone and antimycin A to achieve a steady-state minimal rate, suggesting that these reagents only slowly diffuse into the heart tissue. For cardiac stress measurements, maximal respiration was calculated as the difference between the maximal OCR detected after the addition of 1 mM epinephrine and the minimal rate detected [non-mitochondrial respiration] after addition of rotenone and antimycin A. Evidence suggests that medium acidification rates of 3D tissues are not a reliable indicator of glycolytic lactate production and are more reflective of rates of respiratory CO_2_ production;^76^ therefore, extracellular acidification rates were not reported. All data are mean ± standard error of the mean (SEM) of the individual OCR measurements from each heart assayed over multiple plates as indicated.

### Gas chromatography-mass spectrometry (GC-MS) metabolic profiling study design and sample collection

Five whole flies per biological replicate were collected from each genotype for metabolome characterization. Ages were chosen to capture “adult” 1 week, “aged” 5 week (i.e., median survival for controls), and median survival reported for *VincHE* 11 week.^9^ At each age, the flies were exposed to 5 psi CO_2_, placed into individual tubes at 5 flies per tube, and weighed for normalization. A 9:1 methanol/water mixture was added to each tube at 75 *μ*l/mg fly weight, tubes were placed into liquid nitrogen for snap-freezing, and frozen flies were stored in −80 °C until metabolite extraction was performed.

### Stable isotope tracing study design and sample collection

For each biological replicate, five whole flies per genotype at 1 week were fasted for 12 h prior to tracing experiment by placing them into empty plastic vials with cotton balls overnight. Then, a small aliquot (∼45 *μ*l) of 30 mM [U-^13^C_6_]glucose dissolved in diH_2_O with blue dye was placed onto the side of the vials, which allowed flies to consume immediately. Immediate uniform consumption of the [U-^13^C_6_]glucose mixture was verified using the blue dye indicator in gut tube. At 0.5, 1, 2, and 4 h for preliminary studies in Fig. S3(b), the flies were quickly exposed to 5 psi CO_2_, placed into individual tubes at 5 flies per tube, and weighed so that samples can later be normalized to their weight. Tubes were placed into liquid nitrogen to snap-freeze the flies and stored in −80 °C until metabolite extraction was performed. For Fig. 3, 1 week flies were offered 30 mM [U-^13^C_6_]glucose for 1 h post fasting and collected as depicted above.

### Gas chromatography-mass spectrometry (GC-MS) sample preparation and analysis

Polar metabolites from a pool of five flies per biological replicate were extracted using methanol/water/chloroform. Flies were homogenized in 0.25 ml of −80 °C methanol, 0.25 ml of −20 °C chloroform, and 0.1 ml of 4 °C cold water using a ball mill (Retch) for 2 min at 25 Hz/s for two circles. The volume of the extraction fluid was adjusted to account for differences in fly weight across the samples. The extracts were incubated on a Thermomixer (Eppendorf) at 1400 rpm for 20 min at 4 °C and centrifuged at 16 000 × g for 5 min at 4 °C. 0.2 ml of the upper aqueous phase was evaporated under vacuum at −4 °C using a refrigerated CentriVap Concentrator (Labconco).

Metabolite derivatization was performed using a Gerstel MultiPurpose sampler. Dried metabolites were dissolved in 15 *μ*l of 2% (w/v) methoxyamine hydrochloride (Thermo Scientific) in pyridine and incubated for 60 min at 45 °C. An equal volume of 2,2,2-trifluoro-*N*-methyl-*N*-trimethylsilyl-acetamide (MSTFA) (non-targeted metabolomics study) or *N*-tertbutyldimethylsilyl-*N*-methyltrifluoroacetamide (MTBSTFA) ([^13^C]tracer studies) with 1% tert-butyldimethylchlorosilane (Regis Technologies) was added and incubated further for 30 min at 45 °C.

After derivatization, samples were analyzed by GC-MS using a DB-35MS column (30 m × 0.25 mm i.d. × 0.25 *μ*m, Agilent J&W Scientific) installed in an Agilent 7890A gas chromatograph (GC) interfaced with an Agilent 5977 mass spectrometer (MS) operating under electron impact ionization at 70 eV. The MS source was held at 230 °C and the quadrupole at 150 °C. 1 *μ*l of the derivatized sample was injected, and helium was used as carrier gas at a flow rate of 1 ml/min. For MTBSTFA derivatized samples, the GC oven was held at 100 °C for 1 min, increased to 255 °C at 3.5 °C min^−1^, increased to 320 °C at 15 °C min^−1^, and held at 320 °C for 3 min. For MSTFA measurements, the GC oven was held at 80 °C for 6 min, increased to 300 °C at 6 °C min^−1^, increased to 325 °C at 10 °C min^−1^, and held at 325 °C for 4 min.

Metabolite levels and mass isotopomer distributions were determined by integrating metabolite ion fragments and corrected for natural abundance using MetaboliteDetector software and in-house algorithms. Four biological replicates per genotype and age were analyzed. For non-targeted data, the variation was determined by Principal Component Analysis (PCA), and Fig. 3 depicts relative metabolite abundance normalized to sum of metabolite abundances present per sample. For isotopic labeling experiments, Figs. 4 and S3 are shown as mole percent enrichment (MPE). MPE was calculated as the percent of all atoms within the metabolite pool that are labeled as

$$\sum_{i=1}^{n}\frac{M_{i}\cdot i}{n},$$ (1)

where *n* is the number of carbon atoms in the metabolite and M*i* is the relative abundance of the *i*th mass isotopomer. Relative abundances plotted for Fig. S3 depicts data normalized to controls at 1 week.

### Statistical analysis

All data were checked for Gaussian distribution prior to analysis using D'agnostino-Pearson omnibus normality test. Two-way analysis of variance (ANOVA) was used when comparing two or more groups at more than one condition such as negative geotaxis assay followed by a Bonferroni multiple comparisons post hoc test of significances. Significances are displayed with Bonferroni post-hoc test results denoted over specific comparisons. One-way ANOVA with Dunn's post-test was performed on whole fly metabolite abundance values across genotypes and ages. Lifespan analyses were performed using a log-rank analysis (Mantel-Cox test). In all cases p < 0.05 was taken as significant. All statistical analyses were performed using Prism 5.0a (Graphpad Software, Inc). Specific analyses used are indicated in figure legends. All *in situ* experiments were performed with biological replicates of 15–30 flies unless otherwise indicated and are displayed with average and standard error of the mean (SEM).

## SUPPLEMENTARY MATERIAL

See supplementary material for five figures that outline the methods used in OCR measurements and glucose tracing. These figures also provide additional data for untargeted and traced metabolite data and control fly and cell culture data for rotenone treatments.
